# Objective Assessment of Nuclear and Cortical Cataracts through Scheimpflug Images: Agreement with the LOCS III Scale

**DOI:** 10.1371/journal.pone.0149249

**Published:** 2016-02-18

**Authors:** Alberto Domínguez-Vicent, Ulrika Birkeldh, Laurell Carl-Gustaf, Maria Nilson, Rune Brautaset

**Affiliations:** 1 Optometry Research Group, Department of Optics and Optometry and Vision Science, University of Valencia, Burjasot, Comunidad Valenciana, Spain; 2 Unit of Optometry, Department of Clinical Neuroscience, Karolinska Institutet, Stockholm, Sweden; 3 St. Erik Eye Hospital, Stockholm, Sweden; Rush University Medical Center, UNITED STATES

## Abstract

**Purpose:**

To assess nuclear and cortical opacities through the objective analysis of Scheimpflug images, and to check the correlation with the Lens Opacity Classification System III (LOCS III).

**Methods:**

Nuclear and cortical opacities were graded according to the LOCS III rules after pupil dilation. The maximum and average pixel intensity values along an elliptical mask within the lens nucleus were taken to analyse nuclear cataracts. A new metric based on the percentage of opaque pixels within a region of interest was used to analyse cortical cataracts. The percentage of opaque pixels was also calculated for half, third and quarter areas from the region of interest’s periphery.

**Results:**

The maximum and average intensity values along the nucleus were directly proportional to the LOCS III grade: The larger the LOCS III value, the larger maximum and average intensity ones. These metrics showed a positive and significant correlation with the LOCS grade: The larger the LOCS grade, the higher was percentage of opaque pixels along the cortex within the same mask’s size. This metric showed a significant correlation to the LOCS grade.

**Conclusion:**

The metrics used to assess nuclear opacities showed good correlation with the LOCS III. The percentage of opaque pixels showed to be a useful metric to measure objectively the severity of the cortical opacity. These metrics could be implemented in an algorithm to detect and grade lens opacities automatically and objectively.

## Introduction

The Lens Opacity Classification System III (LOCS III) was developed to overcome the limitations of the LOCS II, i.e., unequal intervals between standards, only one standard for colour grading, only one integer grading, and wide 95% tolerance limit.[1] The LOCS III is the most established subjective system for cataract grading,[2] but still has some limitations because the final value is influenced by slit-lamp settings[3] and the training level of the evaluator.[4] Therefore, objective methods to grade cataracts might reduce these dependences.

Scheimpflug cameras detect back-scattered light coming into the eye to measure densitometric values. This technology has been shown to be superior to optical coherence tomography and ultrasound biomicroscopy techniques to quantify back-scattered light.[5] When it comes to grading the lens opacity, Scheimpflug photography has been shown to be more precise than the LOCS III, which relies on morphological lens changes.[5, 6] Scheimpflug technology also allows the detection of more subtle amounts of cataract progression than the LOCS III [7], which grades lens opacities in steps.[2]

Objective methods have been developed to grade nuclear cataract objectively based on slit-lamp photographs or on Scheimpflug images. An algorithm to grade nuclear cataracts from slit-lamp photographs has been validated, where 95% of the eyes could be graded automatically and objectively.[8] Scheimpflug images have been used to develop an objective metric to quantify nuclear opacities.[2, 9–14] This procedure is based on the pixel intensity measurement within the nucleus area and some studies showed good correlation with the LOCS III.[2, 14, 15]

One study defined two metrics to quantify objectively cortical cataracts: the number of slices with a significant opacity, and an empirical formula.[15] The first one showed to be the best metric to predict the LOCS III cortical grade. Despite these results, the objective metric to grade cortical cataract should be aimed to measure the area of the opaque cortex, which is the procedure used with the LOCS III.[1] All previous studies used Scheimpflug images obtained from the Pentacam HR (Oculus, Germany). However, the Sirius system (Costruzione Strumenti Oftalmici, Florence, Italy) is another Scheimpflug imaging system, and has been used to study anterior segment parameters in healthy[16] and pathological corneas.[17]

In this context, the aims of the present study were the assessment of both nuclear and cortical opacities through objective analysis of Scheimpflug images. A new metric is presented in this study to assess cortical opacities objectively. In both cases the predictability with the LOCS III grading scale was determined. The results obtained in the current study might, in the future, help eye care professionals to grade either nuclear or cortical cataracts objectively.

## Materials and Methods

### Patients

The study population, age range between 42 to 82 years (69.0 ± 9.2 years), was divided into nuclear or cortical group, depending on the opacity location. The nuclear group included 50 patients, and the cortical group included 45. A control group was included for reference purposes and included 39 subjects without any degree of cataract and whose ages ranged between 19 and 30 years (26.24 ± 4.68 years). A young control group was chosen because this population is more likely to have a clear lens. Furthermore, patients with clear lenses with the same age as the population included are difficult, if not impossible, to find.

All patients were diagnosed with cataracts and referred to St. Erik Eye Hospital (Stockholm, Sweden) for cataract extraction and intraocular lens implantation. Prior to surgery an ophthalmic examination including optical biometry, slit lamp examination and non-contact tonometry has been performed on each patient. Both eyes were included in the study if bilateral nuclear or cortical cataract was present. Patients with corneal opacities were excluded from the study since corneal opacities can cast shadows on the lens image that mistakably can be interpreted as cataract. Furthermore, patients with posterior capsular opacities were also excluded since the posterior capsule rarely is seen on a Scheimpflug image, even if the pupil is dilated.

All patients included had either early- or moderate-stage of nuclear or cortical cataract. More mature degrees of cataract were not included since it is very rare in Sweden (surgery is normally undertaken before cataract reach more mature stages). Written informed consent was obtained from all participants. The study was approved by the regional ethics committee in Stockholm (Regionala Etikprovningsnämnden i Stockholm) and performed in accordance with the ethical standards stated in the Declaration of Helsinki.

### Scheimpflug Device

The Sirius system was used to obtain all crystalline lens images. This system combines a single Scheimpflug camera with a Placido disk, and uses a monochromatic slit-light source with a wavelength of 475 nm. This device takes 25 images with 7 degrees steps, and reports a complete 3D analysis of the anterior segment.

### Experimental Procedure

The same experienced ophthalmologist (CGL) graded both nuclear and cortical opacities according to the LOCS III scale 45 minutes after pupil dilation with a combination of topical cyclopentolate 0.75% and phenylephrine 2.50%. To avoid decimal variation or systematic inconsistencies during the LOCS grading, decimal values were only used if the opacity was midway between two standards.[18]

All Scheimpflug images were taken in the same room under dark conditions to avoid undesired reflexes in the images. During each measurement, the subject was seated correctly in front of the instrument by the use of a chin and forehead rest. Three measurements were taken per eye, and the best exam was selected for further analysis.[18] This selection was based on the acquisition image quality, which values were over 95%; and the exam with the maximum number of slices without any shadow cast by the eyelid, eyelash, nose, and so on. Finally, all Scheimpflug images were exported manually for analysis purposes.

### Image Analysis

#### Nuclear cataracts

Nuclear cataracts were analysed through the pixel intensity value along the nucleus with a custom made MATLAB program (Mathworks, Natick, Massachusetts, USA). All pixels in the nucleus were selected with an elliptically shaped region of interest (ROI) because the lens can be estimated as an ellipse located on its centre,[8] and to ensure that the dark zone that separates the nucleus and the cortex was excluded.[2] Furthermore, the ROI was drawn manually for each patient because the nuclear ROI was different from patient to patient, and as a result, a standard mask was not possible to use.

**Fig 1:**
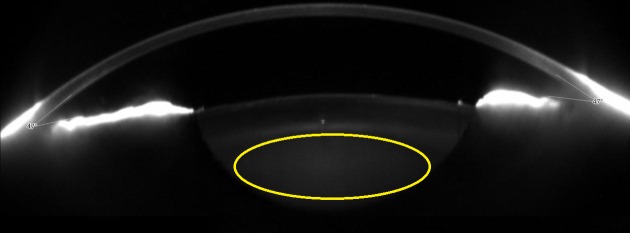
Region of Interest for Measuring the Nuclear Lens Density Superimposed upon the Scheimpflug Image Used.

The ROI size was defined to encompass as much lens nucleus as possible excluding the cortex (Fig 1).[18] Four slices per eye at 45, 90, 135 and 180 degrees were analysed since nuclear cataracts grow homogeneously from the centre to the periphery.[19] To avoid biasing the results, the pixels of artificial reflexes along the nucleus were removed manually. Before retaining these values, the operator was asked to ensure that both the mask and reflex were selected correctly. Finally, the average and maximum values within the ROI were retained for each slice, and the average and maximum values of the four meridians were calculated.

#### Cortical opacities

A custom-made MATLAB program was developed to analyse cortical cataracts through the percentage of opaque pixels along this structure, which is the procedure described in the LOCS III standards.[1] 25 images per eye were analysed in the cataract group because cortical cataract have an asymmetrical progression.[19] In contrast, only 4 images per eye (0, 45, 90 and 135 degrees) were analysed in the control group as cortical transparency was assumed to be homogeneous. The ROI was defined manually to select all pixels within the cortex, taking into account the LOCS III rules.[1] Furthermore, pixels of artificial reflexes located in the cortex were removed manually not to bias the results. To define the ROI, the observer delimited the cortex area, and then a Spline function drew a curve along those points (Fig 1).

**Fig 2:**
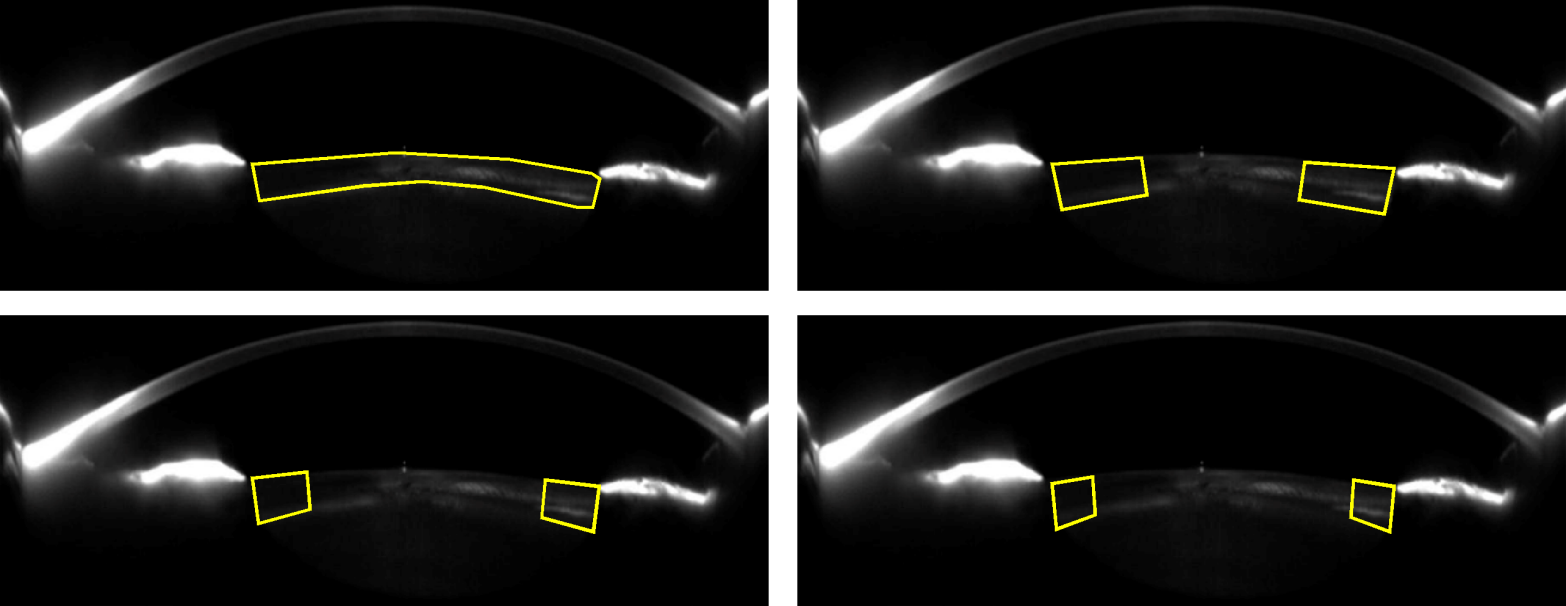
Region of Interest (ROI) for Measuring Cortical Cataracts Superimposed upon the Scheimpflug Image Used. A: ROI to analyse the whole cortex. B: ROI to analyse half cortex. C: ROI to analyse third cortex. D: ROI to analyse quarter cortex.

A threshold value was defined from the clear lens group as the maximum intensity value among all clear eyes to differentiate between clear and opaque pixel. Finally, the percentage of opaque pixels was calculated for the whole cortex (Fig 2, panel A), and for half (Fig 2, panel B), third (Fig 2, panel C) and quarter (Fig 2, panel D) cortex areas from its periphery due to cortical cataracts develop from the periphery towards the centre[20].

### Statistical Analysis

The statistical analysis was performed using SPSS software version 22 (SPSS, Inc. Chicago, IL). The median, maximum and minimum values were reported to describe both populations because the Kolmogorov-Smirnov test revealed that the nuclear opacities and opaque cortical pixels were not normally distributed. The Spearman rank correlation coefficient was used to analyse the relationship between each metric with the LOCS III grade. Statistical significant differences were considered when *P* < 0.05.

## Results

### Nuclear Results

#### Maximum intensity values within the nucleus

Table 1 summarizes the maximum intensity values of the 87 eyes with nuclear cataracts that met the inclusion criteria to proceed with the image analysis. From this table, the clear and 4.0 LOCS grades showed the smallest and largest intensity values in the same slice, respectively. Furthermore, the 2.0 LOCS grade showed lower values than the lowest LOCS score (1.0 LOCS). On the other hand, the reference group showed the narrowest intervals, and the 2.0 and 3.0 LOCS grades had the widest intervals. Finally, the 90 degree slices showed Maximum intensity value among the four meridians within the same LOCS grade.

**Table 1:** Maximum Intensities Summary (Median [Minimum Value–Maximum Value]) within each Slice and the Average of the Four Slices for the Control Group (0.00) and each Lens Opacity Classification System (LOCS) Grade.

	0.00	1.0 LOCS grade	2.0 LOCS grade	3.0 LOCS grade	4.0 LOCS grade
**All Slice**	17.00 [12.00 – 33.00]	28.00 [14.00 – 64.00]	36.50 [16.00 – 103.00]	46.00 [28.00 – 85.00]	59.50 [40.00 – 75.00]
**45 degrees**	15.00 [10.00 – 23.00]	24.50 [14.00 – 61.00]	33.50 [10.00 – 98.00]	41.00 [24.00 – 79.00]	51.00 [38.00 – 65.00]
**90 degrees**	15.00 [11.00 – 22.00]	25.50 [14.00 – 63.00]	35.00 [11.00 – 103.00]	45.50 [25.00 – 85.00]	52.50 [38.00 – 73.00]
**135 degrees**	15.00 [11.00 – 33.00]	24.00 [10.00 – 56.00]	30.50 [15.00 – 95.00]	38.50 [23.00 – 79.00]	49.00 [40.00 – 75.00]
**180 degrees**	14.00 [10.00 – 23.00]	26.00 [14.00 – 64.00]	32.50 [14.00 – 95.00]	41.50 [24.00 – 80.00]	50.50 [38.00 – 61.00]

**Fig 3:**
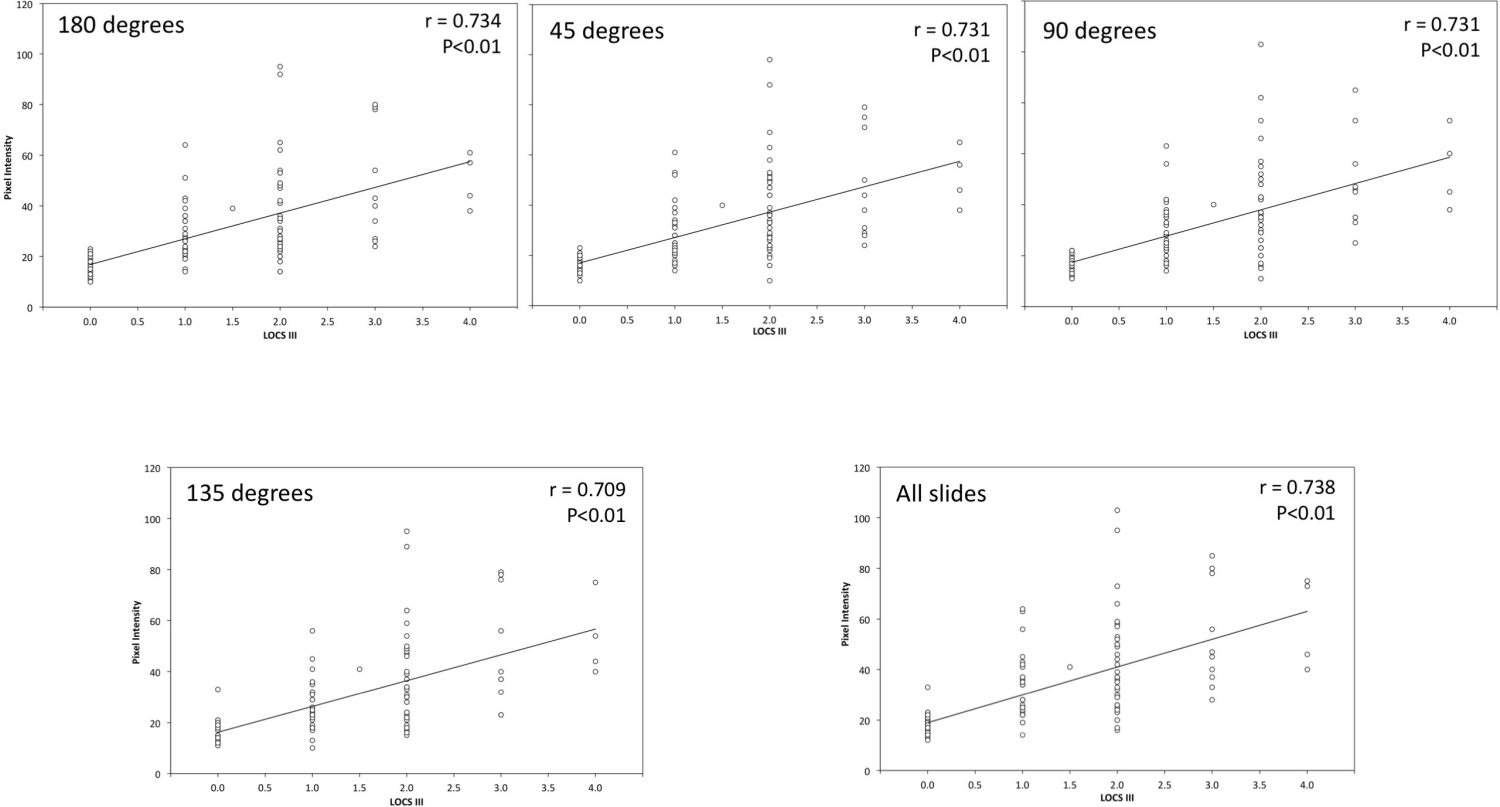
Scatter Plot Representing the Relationship between the Maximum Nuclear Intensity and LOCS III Grade for each Meridian. Top left panel represents the 180 degrees meridian; top centre panel does the 45 degrees meridian; top right panel represents the 90 degrees meridian; bottom left panel does the 135 degrees meridian; and the bottom right panel does the average of the four meridians.

Fig 3 represents the Scatter plot between the nuclear intensity value with its corresponding LOCS grading. The first four panels show the results at 180, 45, 90 and 135 degrees, and the last one represents the average of the four ones. The white points represent the eyes included in the analysis, which positions in the graph were fixed by their corresponding LOCS III grade and the nuclear intensity value. The tendency line was also included to assess the trend between these variables, and the control group was labelled as *‘0*.*0’* because the LOCS III grading ranges from 0.1 to 5.9.

All in all, all panels showed 6 clusters corresponding to each nuclear opacity grade: *0*.*0* and 1.0 LOCS grade, which both included 34 eyes; 1.5 LOCS grade that included 1 eye; and 2.0, 3.0 and 4.0 LOCS grades that encompassed 38, 10 and 4 eyes, respectively. The descriptive statistics showed positive and similar slopes among the tendency lines, where the average of the four slices (Fig 3, bottom right panel) showed the steepest slant. Moreover, significant (P < 0.05) and positive correlations were obtained between the LOCS III grade and maximum pixel value for each comparison.

#### Average intensity values within the nucleus

Table 2 summarizes the average intensity values for each meridian and LOCS III grading. From this table, the 3.0 LOCS grade showed the highest mean value; meanwhile the *0*.*0* grade did the smallest one. On the other hand, the 3.0 LOCS grade resulted with the broadest interval, and the 4.0 LOCS grade with the narrowest. Furthermore, the average nuclear intensity value among each slice and with the same LOCS grade was less than 2 pixel intensity units.

**Table 2:** Averages Intensities Summary (Median [Minimum Value–Maximum Value]) within each Slice and the Average of the Four Slices for the Control Group (0.00) and each Lens Opacity Classification System (LOCS) Grade.

	0.00	1.0 LOCS grade	2.0 LOCS grade	3.0 LOCS grade	4.0 LOCS grade
**All Slice**	7.98 [6.27 – 13.00]	15.59 [7.81 – 41.11]	19.79 [6.93 – 66.60]	23.88 [14.05 – 53.09]	25.55 [22.61 – 26.10]
**45 degrees**	8.26 [6.57 – 13.25]	15.40 [7.80 – 41.80]	21.13 [3.93 – 67.00]	24.22 [12.10 – 52.75]	24.46 [23.02 – 25.44]
**90 degrees**	7.99 [4.20 – 13.71]	15.70 [7.80 – 41.17]	21.02 [5.24 – 63.08]	21.83 [13.89 – 48.80]	27.03 [23.75 – 28.77]
**135 degrees**	8.16 [5.69 – 13.87]	15.78 [3.85 – 38.15]	19.25 [68.36 – 8.48]	24.52 [14.20 – 59.34]	25.21 [22.91 – 27.10]
**180 degrees**	7.99 [3.93 – 12.84]	15.10 [7.53 – 43.30]	19.13 [7.51 – 67.88]	24.95 [13.47 – 56.44]	23.97 [20.75 – 26.13]

**Fig 4:**
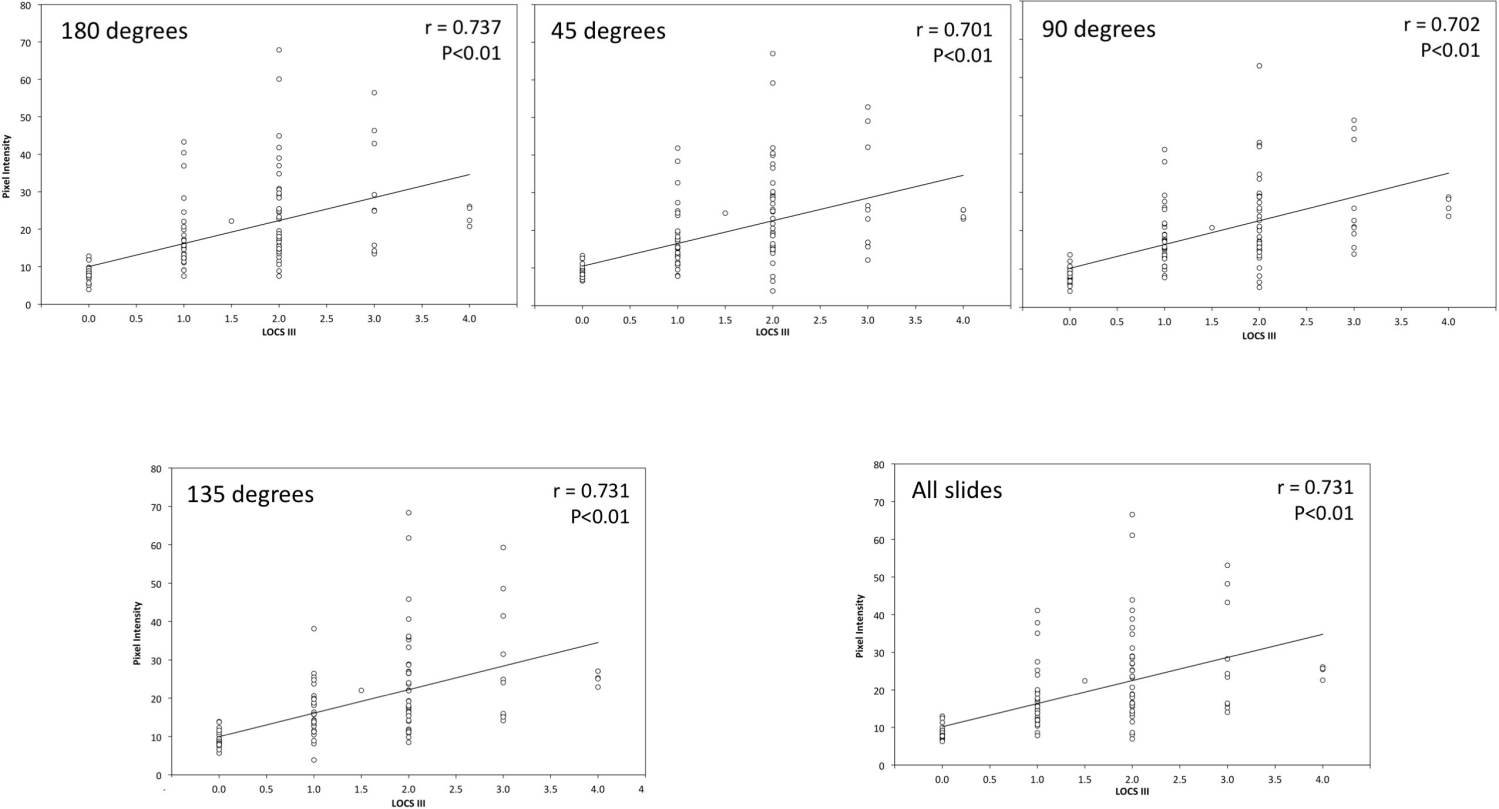
Scatter Plot Representing the Relationship between the Average Nuclear Intensity and LOCS III Grade for each Meridians. Top left panel represents the 180 degrees meridian; top centre panel does the 45 degrees meridian; top right panel represents the 90 degrees meridian; bottom left panel does the 135 degrees meridian; and the bottom right panel does the average of the four meridians.

Fig 4 displays the Scatter plot between the average intensity value in the nucleus and LOCS grading following the same distribution as Fig 3. 6 clusters were obtained in each panel corresponding to *0*.*0*, 1.0 LOCS grades, which both included 34 eyes; and 1.5, 2.0, 3.0, and 4.0 LOCS III grades, which encompassed 1, 38 and 10 eyes, respectively. The statistical analysis revealed a positive and significant correlation (P < 0.05) between the LOCS III and the average intensity values for each slice, and the average of the four. Furthermore, similar slope values were obtained among all slices.

### Cortical Results

#### Threshold value definition

From the 78 clear eyes included, 74 met the inclusion criteria to perform the image analysis. From these eyes, the median value was 25 pixel intensity units, and the minimum and maximum values were 4 and 34 pixel intensity units, respectively. From these results, the threshold pixel value to differentiate between clear and opaque pixels was set to 34 pixel intensity units.

#### Cortical opaque area

Table 3 summarizes the opaque areas of the 81 eyes that finally met the inclusion criteria to perform the image analysis. From this table, the larger the LOCS grading, the largest percentage of opaque pixels along the cortex within the same mask size. This means, the reference group had the smallest percentage of opaque pixels in the cortex and the 3.0 LOCS grade had the largest percentage in the cortex. At the same time, the 0.00 group showed the narrowest intervals, and the 3.0 LOCS grade resulted with the broadest ones for the same mask size. Finally, the percentage of opaque pixels within the same LOCS grade was directly proportional to the mask size. This is, the smaller mask, the smaller percentage of opaque cortex.

**Table 3:** Median and Interval of the Cortex Opacity, Including the Clear one (0.00), as a Function of the Mask Size.

	0.00	0.5 LOCS III	1.0 LOCS III	2.0 LOCS III	3.0 LOCS III
**Whole cortex**	0.34 % [0.00 – 2.63]	4.24 % [0.67 – 31.66]	5.80 % [0.45 – 27.75]	18.88 % [3.46 – 36.13]	23.60 % [2.75 – 44.50]
**Half cortex**	0.00 % [0.00 – 3.63]	1.31 % [0.04 – 27.84]	1.71 % [0.09 – 16.67]	13.53 % [1.50 – 26.50]	17.63 % [1.17 – 34.10]
**Third cortex**	0.00 % [0.00 – 5.33]	0.87 % [0.05 – 26.00]	1.50 % [0.05 – 12.11]	13.30 % [1.34 – 25.11]	14.70 % [1.12 – 28.60]
**Fourth cortex**	0.00 % [0.00 – 7.00]	0.88 % [0.02 – 24.60]	1.48 % [0.04 – 10.80]	13.70 % [1.54 – 24.50]	13.18 % [1.18 – 25.18]

LOCS: Lens Opacity Classification System.

#### Correlations Cortical cataract with LOCS III

**Fig 5:**
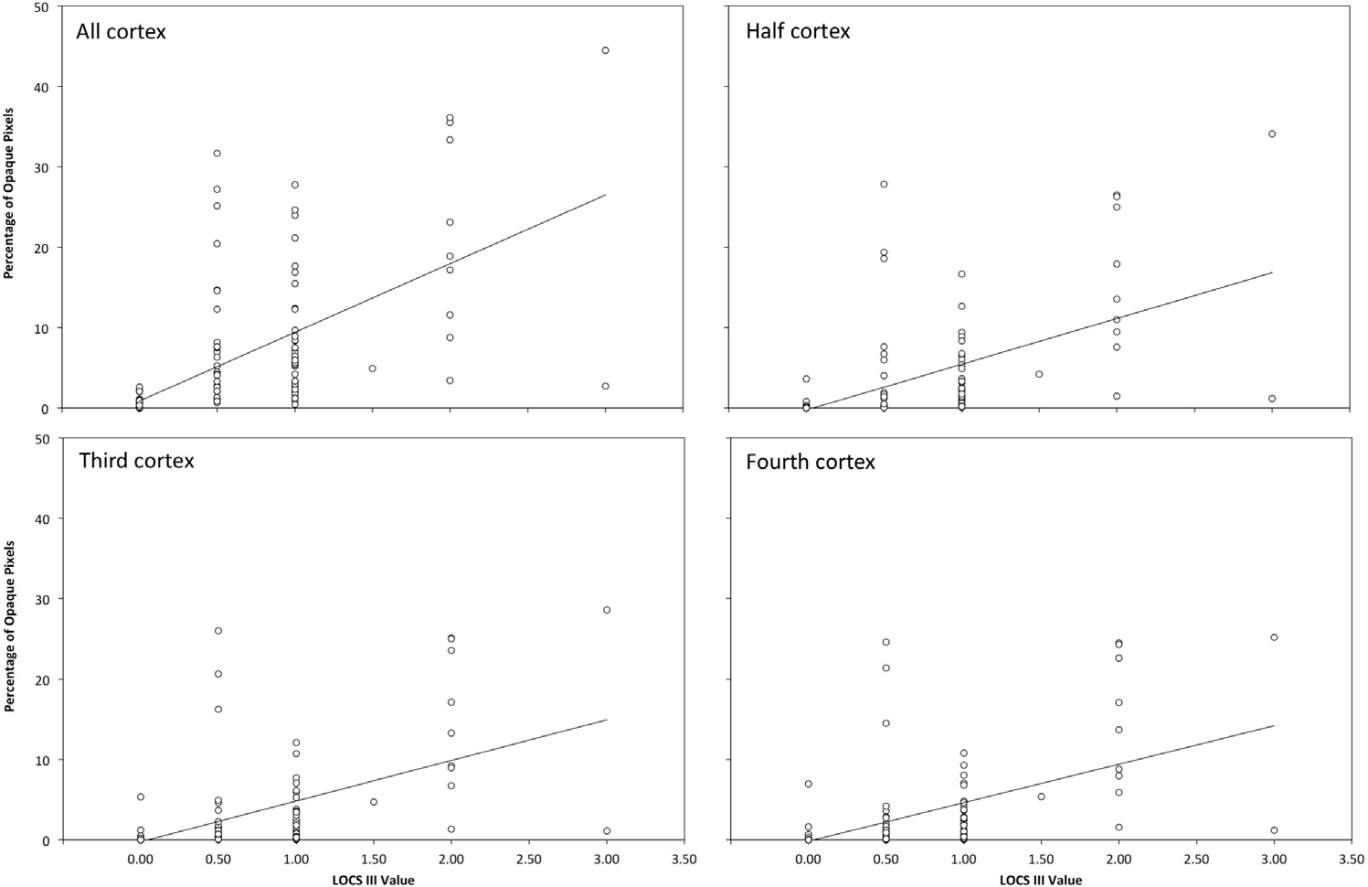
Scatter Plot Representing the Relationship between the Percentage of Opaque Pixels and LOCS III Grade for each Region of Interest Size. Top left panel corresponds to the whole mask size; top right panel does the whole mask size; bottom left panel does third mask size; and bottom right does quarter mask size.

Fig 5 represents the scatter plot between the percentage of opaque pixels and LOCS grading for the whole cortex (top left panel), half cortex (top right panel), third cortex (bottom left panel), and quarter cortex (bottom right panel). The white points represent the eyes analysed, which positions in the graph were fixed by their corresponding LOCS grading and the percentage of opaque cortex. Furthermore, the tendency line was also included to assess the trend between these variables. Values corresponding to the young control group were labelled as ‘*0*.*0’* because the LOCS III grading ranges from 0.1 to 5.9.

Generally speaking, all panels showed 6 clusters corresponding to each cortex grade: the *0*.*0* grade included 74 eyes; the 0.5 and 1.0 LOCS grading encompassed 29 and 40 eyes, respectively; meanwhile the 1.5 LOCS III, 2.0 LOCS grading, and 3.0 LOCS grading included 1, 9, and 2 eyes, respectively. An outlier corresponding to the 3.0 LOCS grading can be distinguished in each figure, which opaque area ranges between 1.0% and 2.0%.

The descriptive statistics revealed a positive slope for each tendency line, which implies that the higher the LOCS III value, the higher the number of opaque pixels. Nevertheless, the whole cortex showed a steeper slope than that obtained for the other analyses. A significant and positive correlation (P < 0.05) was obtained between the LOCS grading and the percentage of opaque pixels. The correlation coefficients were 0.812 for the whole cortex area (Fig 5, top left panel), 0.833 for a half area (Fig 5, top right panel), 0.819 for a third area (Fig 5, bottom left panel), and 0.808 for a quarter cortex area (Fig 5, bottom right panel).

## Discussion

The aims of the present study were the assessment of nuclear and cortical opacities objectively with Scheimpflug images, which were taken with the Sirius system. The nuclear opacities were studied in terms of maximum and average intensity values along the lens nucleus. The percentage of opaque pixels along the cortex is the new objective metric developed to grade cortical opacities.

### Nuclear Cataracts

All in all, the results obtained revealed that the larger the LOCS grading, the larger intensity value. Furthermore, similar average intensity values (Table 2) were obtained among each meridian and the average of the four within the same opacity grade. This is, the nuclear opacity will be the same when only one meridian or the average of the four is analysed. Furthermore, similar correlation values were obtained between the average intensity value among the four slices and the average of the four (Fig 3). These results suggest that only one meridian would be useful to assess the nuclear lens opacity, and the result might be independent of the meridian chosen. Thus, from these results can be concluded that the observer could use one meridian to grade the nuclear opacity in terms of the average intensity value.

A previous study determined the number of meridians needed to optimise the lens nuclear detection using a Scheimpflug camera.[21] These authors used the average opacity along the nucleus and concluded that only the image taken at the meridian with 90 degrees would be useful to estimate the nuclear opacity. Discrepancies between studies could be related to differences in the Scheimpflug camera used. A Nidek EAS-1000 was used in the previous study,[21] and the Sirius device was used in the present one.

The average maximum intensity value of the four meridians showed the largest value (Table 1), similar outcomes were obtained among the four meridians. With regard to correlation coefficients, similar values were obtained among all possible analysis (Fig 4). These results suggest that only one meridian would be useful, but the observer should take into account that the average of the four slices will results in larger opacity values. Beside these results, it should be kept in mind that the maximum intensity value describes the whole nuclear opacity with means of one pixel, and an artificial reflex could falsify the final result. Thus, the average intensity value along the nucleus in the best meridian would be needed to assess the nuclear opacity objectively.

Tables 1 and 2 showed that the larger LOCS III value, the wider interval pixel intensity, which is more noticeable in the average intensity case (Table 1). With this regard, Fig 3 clearly depicts that all LOCS III grades have a pool of points with low pixel intensity values that overlap with the other grades. For example, the results obtained showed that some patients with 3.0 LOCS grading demonstrated similar nuclear opacity as patients graded with *0*.*0* or 1.0 LOCS grading. These results could reflect the difficulty to grade subjectively nuclear opacities, which has been demonstrated to lead to lead inconsistencies between observers.[22] Thus, to overcome this problem, further research should be done to develop new objective grading scales based on objective metrics.

To sum up, the observer should use the average intensity value within the nucleus and one image at 180, 45, 90 or 135 degrees to assess nuclear cataracts objectively with the Sirius. Unfortunately, these conclusions could not be applied to mature cataracts because patients with advanced opacities were not included in the current study. Further studies should assess mature cataracts with this Scheimpflug device.

### Cortical Cataracts

Cortical opacities were assessed objectively in terms of the percentage of opaque pixels along the cortex. As was expected, the larger LOCS grading, the larger percentage of opaque cortex (Table 3). At the same time, similar percentages of opaque pixels were obtained within the same LOCS III grade among different cortex masks. Besides these results, the whole cortex resulted with the largest values, and positive and significant correlations were obtained between the LOCS grading and the percentage of opaque pixels, which were similar among the cortex areas assessed. These similarities revealed that the percentage of opaque pixels is a robust metric to grade cortical cataracts because the correlation did not vary when different cortex areas were taken during the analysis. Finally, due to cortical cataracts grow asymmetrically from the periphery to the central cortex, a procedural approach starting with 1/4, 1/3, 1/2 and in the end the full cortex might be useful to reflect the progression of the cortical cataract.

On the other hand, the steepest slope was obtained when the whole cortex was assessed (Fig 5). This means that differences among LOCS III grades will be greater and more noticeable when the whole cortex is analysed. Therefore, it can be concluded that the whole cortex should be taken into account to analyse early- or moderate-cortical cataract stages. Unfortunately, this conclusion cannot be applied to mature cortical opacities because patients with high opacities were not included in the studies. Further studies assessing this metric in mature cortical cataract would be desirable.

One study developed two metrics to measure the severity of cortical cataracts objectively using the Pentacam.[15] One metric was based on the number of slices containing significant cortical cataract, and the other one consisted on a formula that calculates the percentage of cortical cataract. Their results revealed that the LOCS III cortical grade was best predicted by the number of slices with significant cortical cataracts (r = 0.72, P < 0.001). The correlation between the metric defined in the present study and the LOCS III (r = 0.81, P < 0.01) is similar to that obtained by Ullrich et al. Up to date, there are three different metrics available to measure objectively the severity of cortical cataracts (the percentage of cortical cataracts, the number of slices with significant cortical cataracts, and the percentage of opaque pixels in the cortex). Nevertheless, the last two metrics should be use to grade cortical cataracts because of the strong correlation. Further studies should elucidate whether or not these metrics can be used interchangeably, since both metrics are based on different principles, and different Scheimpflug cameras (Pentacam vs. Sirius) were used to define them.

Pan et al.[23] investigated the relationship between the LOCS III cortex grade and the average lens density, which was measured from Scheimpflug images in 3 dimensions within a diameter of 4 mm. According to these authors, the LOCS III cortical cataract grading did not correlate significantly with the average lens density. Discrepancies between Pan et al results and those obtained in the current study could be related to the metric used to analyse lens opacity. While these authors assessed the lens density in the whole lens, the current study focused the analysis on the extension of the opacity along the cortex. Furthermore, the percentage of opaque pixels follows the standards to grade cortical cataracts.[1] Thus, the last two metrics should be used to grade cortical cataracts objectively.

There is one algorithm validated to grade nuclear cataracts from slit lamp images without any user intervention.[8] With this algorithm 95% of the images, out of more than 5000 slit lamp images, could be diagnosed fully automatically without any user intervention. This study revealed that computer-aid algorithms are useful to grade automatically and objectively nuclear cataracts from slit-lamp images. In fact, machine learning needs 2 seconds to grade nuclear cataracts, while human grading takes about 2 minutes.[24] Currently there is no algorithm to grade cortical cataracts objectively and automatically.

The current study focused on nuclear and cortical opacities with LOCS III grades between 0 and 4 because cataract surgery is commonly performed during the early stages in Sweden. Further studies should use the described metrics to analyse mature cataracts, assess agreement with LOCS III grading and develop an algorithm to detect either the nucleus or cortex automatically, and then grade the opacity objectively.

In conclusion, the best metric to analyse the nuclear opacity objectively would be the average intensity value within the nucleus, with only one image. Furthermore, the novel technique developed to measure cortical cataracts has shown to be a useful procedure to classify objectively the severity of the cortical opacity. Finally, these metrics could be used in conjunction with an algorithm that detects automatically either the nucleus or cortex in order to grade nuclear and cortical cataracts automatically and objectively.

## Supporting Information

S1 Table Cortical Results. (PDF)

S2 Table Nuclear Results. (PDF)
