# Correction: Objective Assessment of Nuclear and Cortical Cataracts through Scheimpflug Images: Agreement with the LOCS III Scale

**DOI:** 10.1371/journal.pone.0152953

**Published:** 2016-03-29

**Authors:** The PLOS ONE staff

The third author’s name is spelled incorrectly. The correct name is: Carl-Gustaf Laurell. The correct citation should be: Domínguez-Vicent A, Birkeldh U, Laurell CG, Nilson M, Brautaset R (2016) Objective Assessment of Nuclear and Cortical Cataracts through Scheimpflug Images: Agreement with the LOCS III Scale. PLoS ONE 11(2): e0149249. doi:10.1371/journal.pone.0149249
