# Supplementary material for: Objective Assessment of Nuclear and Cortical Cataracts through Scheimpflug Images: Agreement with the LOCS III Scale
Source: PLoS One. 2016 Feb 18;11(2):e0149249. doi: 10.1371/journal.pone.0149249 (PMC4758745; doi:10.1371/journal.pone.0149249)
Supplement: S1 Table — (PDF) [file pone.0149249.s001.pdf]

CORTICAL RESULTS

ALL		HALF		THIRD		QUARTER	
Proportion	LOCS III	Proportion	LOCS III	Proportion	LOCS III	Proportion	LOCS III
0.4190	0	0.0000	0	0.0000	0	0.0000	0
0.8918	0	0.0000	0	0.0000	0	0.0000	0
0.8696	0	0.0000	0	0.0000	0	0.0000	0
0.0819	0	0.0000	0	0.0000	0	0.0000	0
0.1387	0	0.0000	0	0.0000	0	0.0000	0
0.2200	0	0.0265	0	0.0403	0	0.0547	0
0.0925	0	0.0170	0	0.0260	0	0.0353	0
0.2423	0	0.0000	0	0.0000	0	0.0000	0
0.0900	0	0.0000	0	0.0000	0	0.0000	0
1.1101	0	0.0000	0	0.0000	0	0.0000	0
0.1412	0	0.0000	0	0.0000	0	0.0000	0
0.0000	0	0.0000	0	0.0000	0	0.0000	0
0.3151	0	0.0000	0	0.0000	0	0.0000	0
2.0969	0	0.0000	0	0.0000	0	0.0000	0
1.1226	0	0.0000	0	0.0000	0	0.0000	0
2.2271	0	0.0000	0	0.0000	0	0.0000	0
2.6309	0	0.1430	0	0.2191	0	0.3026	0
0.2745	0	0.0000	0	0.0000	0	0.0000	0
0.6545	0	0.8273	0	1.2277	0	1.6231	0
0.4226	0	0.0000	0	0.0000	0	0.0000	0
0.3429	0	0.0084	0	0.0133	0	0.0186	0
0.8326	0	0.0000	0	0.0000	0	0.0000	0
0.7958	0	0.0025	0	0.0036	0	0.0048	0
0.3999	0	0.0000	0	0.0000	0	0.0000	0
0.2059	0	0.0000	0	0.0000	0	0.0000	0
0.6784	0	0.0075	0	0.0112	0	0.0149	0
0.0224	0	0.0000	0	0.0000	0	0.0000	0
2.0343	0	3.6383	0	5.3347	0	6.9530	0
0.3928	0	0.2999	0	0.4722	0	0.6574	0
0.6153	0	0.0000	0	0.0000	0	0.0000	0
0.0000	0	0.0000	0	0.0000	0	0.0000	0
0.0000	0	0.0000	0	0.0000	0	0.0000	0
0.2525	0	0.0000	0	0.0000	0	0.0000	0
0.5342	0	0.0093	0	0.0144	0	0.0202	0
0.0070	0	0.0137	0	0.0206	0	0.0277	0
0.0000	0	0.0000	0	0.0000	0	0.0000	0
0.0000	0	0.0000	0	0.0000	0	0.0000	0
0.0993	0	0.0277	0	0.0420	0	0.0582	0
0.1155	0	0.0104	0	0.0155	0	0.0209	0
0.8264	0	0.0000	0	0.0000	0	0.0000	0
0.5106	0	0.0055	0	0.0082	0	0.0109	0
0.3096	0	0.0476	0	0.0700	0	0.0933	0
0.4248	0	0.0910	0	0.1326	0	0.1752	0
0.4643	0	0.0138	0	0.0202	0	0.0268	0
0.6910	0	0.0105	0	0.0159	0	0.0217	0

0.8239	0	0.0000	0	0.0000	0	0.0000	0
0.0000	0	0.0000	0	0.0000	0	0.0000	0
0.0351	0	0.0459	0	0.0693	0	0.0934	0
0.7199	0	0.0000	0	0.0000	0	0.0000	0
0.2236	0	0.0181	0	0.0287	0	0.0396	0
1.0101	0	0.3342	0	0.4987	0	0.6674	0
0.8163	0	0.0000	0	0.0000	0	0.0000	0
0.0000	0	0.0000	0	0.0000	0	0.0000	0
0.0000	0	0.0000	0	0.0000	0	0.0000	0
0.1713	0	0.0000	0	0.0000	0	0.0000	0
0.3060	0	0.0000	0	0.0000	0	0.0000	0
0.3426	0	0.0000	0	0.0000	0	0.0000	0
0.0895	0	0.0000	0	0.0000	0	0.0000	0
1.0933	0	0.0000	0	0.0000	0	0.0000	0
0.6235	0	0.0000	0	0.0000	0	0.0000	0
2.0619	0	0.0079	0	0.0122	0	0.0168	0
0.7041	0	0.0000	0	0.0000	0	0.0000	0
0.0000	0	0.0000	0	0.0000	0	0.0000	0
0.3361	0	0.0000	0	0.0000	0	0.0000	0
0.9881	0	0.0000	0	0.0000	0	0.0000	0
0.3352	0	0.0000	0	0.0000	0	0.0000	0
0.0000	0	0.0000	0	0.0000	0	0.0000	0
0.0744	0	0.1392	0	0.2122	0	0.2877	0
0.5030	0	0.0000	0	0.0000	0	0.0000	0
0.4267	0	0.0133	0	0.0217	0	0.0308	0
0.8792	0	0.0456	0	0.0000	0	0.0000	0
0.9198	0	0.0000	0	0.0000	0	0.0000	0
0.1272	0	0.0000	0	0.0000	0	0.0000	0
0.3458	0	0.0000	0	0.0000	0	0.0000	0
7.0163	0.5	0.5217	0.5	0.3691	0.5	0.3965	0.5
4.4671	0.5	0.4269	0.5	0.5420	0.5	0.6860	0.5
2.7805	0.5	1.5885	0.5	1.4281	0.5	1.3773	0.5
14.6891	0.5	4.0246	0.5	1.5577	0.5	1.2016	0.5
7.5314	0.5	1.5061	0.5	1.6163	0.5	2.0389	0.5
20.4133	0.5	7.6279	0.5	4.6255	0.5	3.5893	0.5
8.1986	0.5	1.8951	0.5	0.8677	0.5	0.8628	0.5
2.7277	0.5	0.3384	0.5	0.3655	0.5	0.3541	0.5
2.6402	0.5	1.4013	0.5	1.0373	0.5	1.0827	0.5
6.3476	0.5	1.3169	0.5	1.5455	0.5	1.5777	0.5
1.0319	0.5	0.2225	0.5	0.3349	0.5	0.4541	0.5
1.1852	0.5	0.1160	0.5	0.1668	0.5	0.2246	0.5
1.2942	0.5	0.1180	0.5	0.0919	0.5	0.0232	0.5
27.1941	0.5	19.3490	0.5	20.6533	0.5	21.4097	0.5
7.6134	0.5	1.1970	0.5	0.5921	0.5	0.4898	0.5
0.6734	0.5	0.2884	0.5	0.4303	0.5	0.4971	0.5
2.6427	0.5	0.4140	0.5	0.1073	0.5	0.1435	0.5
3.3454	0.5	1.9320	0.5	1.7983	0.5	1.6796	0.5
12.2994	0.5	6.0018	0.5	3.6750	0.5	2.8533	0.5
31.6625	0.5	27.8435	0.5	26.0021	0.5	24.6078	0.5
25.1384	0.5	18.5837	0.5	16.2388	0.5	14.4943	0.5
1.3102	0.5	0.0668	0.5	0.0737	0.5	0.0861	0.5
0.8642	0.5	0.0441	0.5	0.0586	0.5	0.0807	0.5
5.2433	0.5	0.2098	0.5	0.3096	0.5	0.4171	0.5
2.0747	0.5	1.7148	0.5	2.2443	0.5	2.7545	0.5
4.2413	0.5	0.0559	0.5	0.0873	0.5	0.1028	0.5
4.1210	0.5	1.3942	0.5	1.2022	0.5	1.2209	0.5
2.1288	0.5	0.5051	0.5	0.6926	0.5	0.8884	0.5
14.5927	0.5	6.7407	0.5	4.9093	0.5	4.1765	0.5
6.4149	1	2.1448	1	2.4413	1	2.6748	1
23.9277	1	9.3886	1	5.9203	1	4.8325	1
27.7593	1	16.6738	1	12.1197	1	10.8078	1
21.1465	1	6.4997	1	3.8071	1	3.6538	1
15.4662	1	8.9035	1	7.6829	1	7.0734	1
17.6623	1	12.6666	1	10.7228	1	9.3017	1
4.2396	1	1.7115	1	1.7118	1	2.0173	1
7.0157	1	0.9127	1	0.3490	1	0.4045	1
7.4716	1	3.6803	1	3.5823	1	3.7235	1
2.6969	1	1.2293	1	1.2647	1	1.2286	1
5.2057	1	1.2196	1	1.1396	1	1.1057	1
2.3717	1	2.5339	1	3.4916	1	4.1776	1
6.4506	1	2.1852	1	2.1030	1	2.4496	1
3.2736	1	0.4320	1	0.3357	1	0.4149	1
16.8610	1	3.2018	1	0.7055	1	0.5568	1
24.6221	1	8.3629	1	3.4275	1	1.7343	1
7.5157	1	0.6457	1	0.2077	1	0.2378	1
8.3581	1	6.0001	1	6.1561	1	6.8052	1
2.7051	1	0.1033	1	0.0489	1	0.0440	1

5.4232	1	0.7894	1	0.5084	1	0.5013	1
5.5995	1	2.5286	1	2.3121	1	2.3046	1
0.9461	1	0.1404	1	0.2167	1	0.3007	1
9.6461	1	1.6299	1	0.4990	1	0.3320	1
8.4272	1	1.7227	1	0.4739	1	0.2021	1
8.9345	1	1.4056	1	1.2219	1	1.0745	1
5.4539	1	0.4268	1	0.2693	1	0.1628	1
5.6532	1	5.3991	1	7.0875	1	8.0474	1
3.3879	1	1.4042	1	0.8873	1	0.7713	1
2.0185	1	0.5658	1	0.7848	1	1.0261	1
6.0092	1	2.4410	1	2.5650	1	2.8720	1
1.6897	1	0.5965	1	0.4074	1	0.3366	1
1.2452	1	0.2692	1	0.2711	1	0.3215	1
12.4064	1	4.9317	1	3.0584	1	2.6162	1
12.2921	1	6.7609	1	5.2432	1	4.6290	1
2.3388	1	1.4572	1	2.1304	1	2.7332	1
5.9802	1	3.3440	1	3.4572	1	3.7355	1
0.4581	1	0.0957	1	0.1461	1	0.1995	1
3.0032	1	0.1995	1	0.1470	1	0.1834	1
3.3562	1	1.7551	1	1.8345	1	1.8237	1
1.2444	1	0.1975	1	0.3018	1	0.3654	1
4.9239	1.5	4.2266	1.5	4.7204	1.5	5.3701	1.5
11.5708	2	7.5857	2	6.7352	2	5.8823	2
33.3359	2	24.9904	2	23.5857	2	22.5999	2
23.0907	2	17.9069	2	17.1462	2	17.0929	2
35.5429	2	13.5304	2	25.1198	2	24.5183	2
36.1346	2	10.9984	2	25.0131	2	24.3139	2
8.7671	2	1.4822	2	9.2287	2	8.7758	2
18.8786	2	9.4702	2	13.2751	2	13.6976	2
17.1610	2	26.4929	2	8.9673	2	7.9722	2
3.4606	2	26.2916	2	1.3412	2	1.5412	2
44.4705	3	34.0987	3	1.1249	3	25.1887	3
2.7554	3	1.1765	3	28.6007	3	1.1884	3
