# Supplementary material for: Objective Assessment of Nuclear and Cortical Cataracts through Scheimpflug Images: Agreement with the LOCS III Scale
Source: PLoS One. 2016 Feb 18;11(2):e0149249. doi: 10.1371/journal.pone.0149249 (PMC4758745; doi:10.1371/journal.pone.0149249)
Supplement: S2 Table — (PDF) [file pone.0149249.s002.pdf]

NUCLEAR RESULTS

All Slides			45 degrees			
Average Int	Max Int	LOCS III	Average Int	Max Int	LOCS III	Average Int
12.805552	22	1	13.3736376	20	1	12.6491309
14.4611208	28	1	13.3796098	23	1	15.2464034
13.748203	26	1	13.5697377	23	1	14.0723716
37.8314207	56	1	38.3026058	53	1	37.9949664
41.1152772	63	1	41.7928637	61	1	41.1693089
18.9515507	34	1	17.6249934	31	1	17.6343473
27.4358999	43	1	27.316039	42	1	27.5864452
11.6924787	19	1	10.7613922	18	1	10.7222186
16.9891297	28	1	17.7193166	28	1	18.123119
35.0478339	64	1	32.646147	52	1	29.2080662
17.5032363	36	1	17.0681251	34	1	17.4917148
17.7939501	34	1	18.1771898	32	1	17.2819954
12.7553926	25	1	11.4975769	25	1	13.5018665
23.9493511	41	1	23.9393662	39	1	26.0635651
12.1510531	22	1	12.9567416	21	1	13.0990799
11.7140493	22	1	13.5460615	20	1	12.9656724
14.6767929	28	1	14.1513662	25	1	15.0799802
17.7987693	28	1	17.5688612	25	1	17.0840869
25.2069513	45	1	25.1736079	31	1	25.5774576
14.5187757	23	1	14.0786025	23	1	13.5984466
15.8506583	34	1	15.7479192	24	1	15.3761977
19.7229776	42	1	19.3328573	28	1	19.0108849
8.56495612	22	1	9.52789912	16	1	9.80214379
15.6006298	35	1	7.99972792	17	1	20.9775112
18.9135826	35	1	19.7760112	34	1	18.929023
10.4458678	19	1	10.7488758	16	1	8.22289157
15.5875884	24	1	15.1536875	23	1	15.636857
20.0646895	37	1	24.6140301	37	1	21.7622304
18.9365074	37	1	18.2924302	33	1	21.9609264
7.81624609	14	1	7.79831261	14	1	7.79481833
13.8405182	25	1	15.3437138	21	1	12.6567572
13.8186564	25	1	15.4636923	21	1	15.7792735
11.9615896	22	1	12.6984619	22	1	10.6179713
10.785025	22	1	11.2295845	17	1	10.6828167
22.3876544	41	1.5	24.5561524	40	1.5	20.7804453
12.9257205	26	2	15.367718	23	2	12.9301476
18.9265517	36	2	19.5057691	34	2	17.3486593
15.54101	30	2	15.7421203	28	2	15.1758504
43.8958958	66	2	41.8560328	63	2	43.0199719
41.1488472	73	2	39.8927842	69	2	42.2274215
18.6041171	35	2	19.0490076	33	2	20.9124227
28.4780782	49	2	29.5467739	47	2	28.7890148
31.1117469	58	2	32.535436	58	2	25.8315975
20.6669623	39	2	24.8870899	39	2	20.0286977
23.3318949	42	2	15.482111	26	2	23.7622273
16.2556579	30	2	18.5462306	28	2	14.8957525
17.9385616	32	2	21.7639181	31	2	21.1381655

61.0176768	103	2	59.1352846	98	2	63.0600371
16.6004424	23	2	15.6793799	20	2	17.0805361
16.5605465	29	2	18.5650782	29	2	18.3263603
11.4867348	25	2	11.2908544	22	2	10.2586243
13.9305726	26	2	14.6707065	26	2	13.5128447
34.7951951	50	2	37.589768	50	2	33.4629847
66.5804318	95	2	66.9935233	88	2	63.0800173
36.5457131	53	2	36.5106245	47	2	34.7146697
13.7012993	20	2	14.3890089	19	2	14.8922867
25.266923	52	2	30.2201399	52	2	29.3845944
28.9873723	59	2	28.9476496	51	2	29.7437057
16.2640313	23	2	15.9290242	23	2	16.0936226
26.9367225	46	2	28.7974684	44	2	27.342674
27.0422056	50	2	27.0352285	49	2	29.0239428
14.3312025	29	2	13.9548109	27	2	14.3987842
8.07085412	20	2	3.93104273	10	2	6.57872839
28.8681434	37	2	28.3186707	36	2	28.8518401
38.875638	57	2	40.4420328	53	2	41.9432608
6.9338031	16	2	6.49334738	16	2	5.24055892
15.8225557	24	2	16.4182393	24	2	16.6923977
16.4876613	26	2	14.9667392	24	2	15.6971369
25.1390992	44	2	25.4909626	44	2	25.2256469
18.7743405	33	2	20.5062879	33	2	18.3779537
23.5974274	37	2	23.1048175	37	2	22.6892174
25.1245471	37	2	25.0826981	36	2	25.907858
8.66458426	17	2	7.7564071	16	2	8.20492738
23.4480347	40	3	26.5132937	50	3	21.0234005
15.2440419	28	3	52.7575758	71	3	13.8970463
43.2679839	78	3	15.7350335	24	3	46.6678981
48.170797	85	3	25.4675789	44	3	48.8081117
28.2574276	56	3	16.8290218	31	3	25.8236952
53.0928721	80	3	12.0850036	29	3	43.8307475
16.2457314	47	3	22.9882558	38	3	20.7957805
24.3208517	45	3	15.6536977	28	3	22.6372845
16.4458248	37	3	42.0463659	75	3	19.1931063
14.0595545	33	3	48.9899723	79	3	15.5622855
25.4402154	75	4	23.0216044	38	4	23.7591153
26.0890815	73	4	25.4030677	46	4	25.8566569
22.6123794	40	4	23.5367761	56	4	28.7722808
25.678972	46	4	25.4364724	65	4	28.2142781
9.21350682	33	0	9.94471219	21	0	4.20200651
6.80929605	16	0	7.90943233	16	0	8.77608441
9.34379867	16	0	8.47214201	18	0	6.62553579
7.67300404	18	0	8.41779499	14	0	7.99038696
8.16099774	16	0	7.99534622	13	0	7.65390431
7.78500395	18	0	7.58063188	12	0	7.35041607
7.60302638	15	0	7.16711935	15	0	8.74931602

7.47623221	17	0	8.59922249	19	0	7.14589555
7.6603032	19	0	8.81977381	15	0	8.84757818
8.59909551	17	0	8.25276921	13	0	6.23895074
8.47403615	20	0	7.73597377	16	0	9.35024926
7.48310005	19	0	9.40016332	20	0	10.4760386
9.34369514	21	0	10.2616047	21	0	7.42645997
9.9987783	23	0	7.19806538	17	0	9.91465363
7.87077805	19	0	9.0266873	17	0	7.74553966
9.02278412	22	0	7.8652417	13	0	7.74591963
7.91778932	13	0	8.19453458	14	0	9.30687529
8.09540855	14	0	8.11271149	14	0	8.63291
8.55917026	18	0	9.22117523	15	0	7.13065813
9.17157112	18	0	8.44871395	15	0	7.96524941
8.34054599	15	0	6.65657976	12	0	7.00915253
7.9080811	15	0	7.47955001	14	0	9.69038196
6.74959176	12	0	7.40798389	13	0	5.76285727
6.55309309	14	0	8.85199392	16	0	8.74147696
8.00482903	14	0	6.57408526	10	0	13.7186628
8.61333996	18	0	8.42977566	14	0	6.85877522
6.85673793	14	0	12.5266642	20	0	8.17978673
7.93824794	16	0	13.2583919	23	0	5.53025362
13.0047216	22	0	6.84778423	14	0	8.88630805
12.9583964	23	0	7.95259246	14	0	12.042655
7.33755765	15	0	7.51136293	13	0	10.6680314
7.59294945	17	0	8.2705493	16	0	
6.27034929	14	0	12.5858086	19	0	
7.67444676	17	0	11.1270482	20	0	
12.4502207	21	0				
11.2940932	22	0				

90 degrees			135 degrees			180 degrees	
Max Int	LOCS III	Average Int	Max Int	LOCS III	Average Int	Max Int	LOCS III
22	1	12.7747438	21	1	12.4246956	21	1
28	1	14.2824619	24	1	14.9360079	26	1
26	1	14.0666904	24	1	13.2840123	20	1
56	1	38.1520822	56	1	36.8760284	51	1
63	1	20.6549862	32	1	40.383659	51	1
32	1	26.5099689	41	1	19.8918759	34	1
42	1	12.7133758	19	1	28.3311465	43	1
18	1	16.2546721	23	1	12.5729284	19	1
25	1	18.2586803	35	1	15.8594111	27	1
38	1	18.481538	32	1	43.2892885	64	1
33	1	13.5035826	23	1	17.1944251	36	1
32	1	23.7555896	36	1	17.235077	34	1
25	1	10.4492869	18	1	12.5185445	22	1
41	1	11.2058557	18	1	22.0388834	36	1
22	1	14.202381	25	1	12.0991041	22	1
22	1	18.1355768	24	1	9.13860768	15	1
25	1	25.4970707	45	1	15.2734443	28	1
28	1	15.7373535	23	1	18.4065522	27	1
42	1	16.3380121	26	1	24.579669	39	1
23	1	19.841192	29	1	14.6607003	23	1
25	1	3.8535741	10	1	15.9405041	34	1
29	1	18.7948516	35	1	20.7069761	42	1
18	1	19.9391685	31	1	11.0762075	22	1
33	1	11.5154452	17	1	14.6304285	29	1
35	1	15.8308607	22	1	17.0101275	31	1
16	1	24.8083244	36	1	11.2962588	19	1
24	1	19.7396181	29	1	15.7289482	23	1
36	1	8.1412594	13	1	9.07417312	26	1
37	1	13.8695399	25	1	15.753055	24	1
14	1	11.5402101	23	1	7.530594	14	1
20	1	11.271974	18	1	13.492062	21	1
25	1	8.89840542	18	1	12.4914497	24	1
18	1	22.0624803	41	1.5	13.2579515	22	1
17	1	11.8932093	22	2	12.3292935	22	1
40	1.5	19.3260342	28	2	22.1515396	39	1.5
23	2	15.5205188	30	2	11.511807	26	2
36	2	45.8588758	64	2	19.525744	31	2
30	2	40.6819206	64	2	15.7255504	28	2
66	2	17.3278259	34	2	44.8487027	62	2
73	2	26.477627	47	2	41.7932625	65	2
35	2	35.2752068	54	2	17.1272124	35	2
48	2	22.1234666	39	2	29.0988972	49	2
55	2	21.9999279	31	2	30.8047475	54	2
34	2	19.1846232	30	2	18.7517667	30	2
42	2	17.7868699	24	2	22.6783344	35	2
23	2	61.796778	95	2	15.4601451	28	2
32	2	16.5799523	22	2	14.2829804	24	2

103	2	11.2949365	19	2	60.0786074	92	2
23	2	11.8486154	17	2	17.0619013	22	2
29	2	14.0123342	21	2	18.0558108	25	2
16	2	33.3442826	39	2	12.5488451	25	2
26	2	68.363445	89	2	13.5264051	20	2
42	2	35.9784423	48	2	34.7837449	41	2
82	2	11.4953023	18	2	67.8847414	95	2
43	2	10.9338572	28	2	38.9791161	53	2
20	2	28.8719281	59	2	14.0285992	18	2
48	2	16.3588732	21	2	30.5291004	47	2
52	2	27.0443888	46	2	28.3862058	53	2
20	2	26.9113334	49	2	16.6746052	22	2
42	2	14.2710973	23	2	24.5623588	34	2
50	2	11.160756	18	2	25.1983178	48	2
29	2	28.7050044	33	2	14.7001177	27	2
15	2	36.1826392	50	2	10.6128894	20	2
37	2	8.48790817	15	2	29.5970583	35	2
57	2	15.3889989	22	2	36.9346192	49	2
11	2	17.7269542	24	2	7.51339792	14	2
23	2	26.559464	40	2	14.7905871	23	2
26	2	18.1265032	30	2	17.5598148	24	2
43	2	23.7207745	37	2	23.2803233	42	2
32	2	24.049042	34	2	18.0866172	30	2
35	2	9.79574083	16	2	24.8749005	36	2
37	2	24.926063	37	3	25.4585902	35	2
17	2	15.6608447	23	3	8.90126173	14	2
35	3	41.5075234	76	3	13.7885518	34	3
25	3	48.6093198	79	3	13.4735079	27	3
73	3	31.5176068	56	3	25.0516389	43	3
85	3	59.3450219	78	3	24.8544196	40	3
46	3	14.2046804	23	3	15.764579	26	3
56	3	24.1269045	40	3	42.8501484	78	3
47	3	15.9726194	37	3	46.2757843	79	3
45	3	15.1174211	32	3	29.175115	54	3
35	3	22.913607	40	4	56.438143	80	3
33	3	25.323699	44	4	14.2474312	24	3
38	4	27.103114	75	4	20.7551906	38	4
45	4	25.0967554	54	4	26.1324643	44	4
60	4				22.3486908	61	4
73	4				25.60882	57	4
		10.348861	33	0			
		8.03466526	12	0			
13	0	11.0281618	15	0	7.34694728	19	0
15	0	7.9624793	13	0	7.09108009	13	0
17	0	7.71336697	14	0	8.22714981	16	0
16	0	7.91685736	18	0	7.63185905	14	0
12	0	7.75732347	15	0	8.52244205	14	0
14	0	8.67955738	17	0	7.44280827	12	0
19	0	7.81365778	15	0	7.42024585	12	0

14	0	9.02743337	15	0	6.70783605	13	0
20	0	8.06124667	15	0	5.47901648	12	0
11	0	8.25179712	19	0	9.40327933	17	0
19	0	8.93779956	21	0	8.73455056	16	0
22	0	9.40203608	20	0	7.70567855	15	0
13	0	9.04933542	17	0	9.68656842	21	0
22	0	8.51508258	18	0	9.85543385	23	0
13	0	8.06858297	12	0	7.80925145	19	0
15	0	9.36096837	18	0	8.63471296	13	0
16	0	8.55915603	13	0	7.99179293	12	0
15	0	7.36744825	13	0	7.99628253	13	0
12	0	7.64162551	12	0	9.01708157	14	0
14	0	5.68314387	11	0	9.59907791	18	0
12	0	8.72819036	14	0	8.04818198	13	0
18	0	7.62207247	12	0	5.56950363	11	0
11	0	8.23327124	14	0	5.08442908	12	0
16	0	7.38626766	11	0	8.87398936	14	0
22	0	13.8714361	20	0	8.2889115	14	0
14	0	13.7723271	21	0	7.19547149	13	0
17	0	7.93663462	13	0	11.9021233	22	0
13	0	6.57082636	13	0	11.8444702	20	0
17	0	8.10326163	14	0	7.70703653	15	0
21	0	7.84796207	12	0	7.66859224	13	0
22	0	12.3304108	20	0	3.93651897	10	0
		11.6075657	19	0	5.69296762	10	0
					12.8420085	21	0
					11.7737274	18	0
